# A regression analysis of gene expression in ES cells reveals two gene classes that are significantly different in epigenetic patterns

**DOI:** 10.1186/1471-2105-12-S1-S50

**Published:** 2011-02-15

**Authors:** Sung-Joon Park, Kenta Nakai

**Affiliations:** 1 Human Genome Center, Institute of Medical Science, University of Tokyo, Japan

## Abstract

**Background:**

To understand the gene regulatory system that governs the self-renewal and pluripotency of embryonic stem cells (ESCs) is an important step for promoting regenerative medicine. In it, the role of several core transcription factors (TFs), such as Oct4, Sox2 and Nanog, has been intensively investigated, details of their involvement in the genome-wide gene regulation are still not well clarified.

**Methods:**

We constructed a predictive model of genome-wide gene expression in mouse ESCs from publicly available ChIP-seq data of 12 core TFs. The tag sequences were remapped on the genome by various alignment tools. Then, the binding density of each TF is calculated from the genome-wide bona fide TF binding sites. The TF-binding data was combined with the data of several epigenetic states (DNA methylation, several histone modifications, and CpG island) of promoter regions. These data as well as the ordinary peak intensity data were used as predictors of a simple linear regression model that predicts absolute gene expression. We also developed a pipeline for analyzing the effects of predictors and their interactions.

**Results:**

Through our analysis, we identified two classes of genes that are either well explained or inefficiently explained by our model. The latter class seems to be genes that are not directly regulated by the core TFs. The regulatory regions of these gene classes show apparently distinct patterns of DNA methylation, histone modifications, existence of CpG islands, and gene ontology terms, suggesting the relative importance of epigenetic effects. Furthermore, we identified statistically significant TF interactions correlated with the epigenetic modification patterns.

**Conclusions:**

Here, we proposed an improved prediction method in explaining the ESC-specific gene expression. Our study implies that the majority of genes are more or less directly regulated by the core TFs. In addition, our result is consistent with the general idea of relative importance of epigenetic effects in ESCs.

## Background

Embryonic stem cells (ESCs) derived from blastocysts are self-renewal and pluripotent [1-3]. To understand the gene regulatory system in ESCs is an important step for uncovering the process of cell fate determination and for promoting regenerative medicine. Considerable recent evidence indicates that several transcription factors (TFs), so-called core TFs, are indispensable to maintain the pluripotency [4,5]. Some of the core TFs reprogram somatic cells back to pluripotent states [6,7]. These observations suggest that the regulatory network of TFs apparently governs the self-renewal and pluripotency [8,9]. On the other hand, many studies have reported that other TFs can functionally substitute for the core TFs [10-13], suggesting that there still exist additional or alternative TFs unrevealed in the network. Epigenetic modifications are also essential for ESCs [14,15]. Their involvement in the maintenance of the pluripotency is still not well clarified.

To understand the regulatory mechanism underlying in ESCs, a number of methods have been developed. In particular, massive parallel sequencing [9,16-19] and various *in silico* approaches [8,9,20,21] have yielded comprehensive recent advances in our understanding. In this study, we focus on predicting the gene expression in ESCs with the massive parallel sequencing data. Although a previous study successfully applied a regression model to the prediction [21], the model is based on a generalized weighting scheme to prepare predictors (explanatory variables). Intuitively, such weighting scheme cannot reflect the nature of the spatial rearrangement of TF-binding.

Here, we propose a density-based approach that uses the genome-wide bona fide TF binding sites. First, a publicly available ChIP-seq data [9] is reanalyzed. Then, density profiles of TFs estimated from the ChIP-seq data are adopted as predictors in a simple linear regression model to predict the genome-wide gene expression. Predictors are also combined with epigenetic data, such as H3K4me3, H3K27me3, DNA methylation, and CpG island [16,17]. Furthermore, we analyze the regulatory effects of TFs, epigenetic states, and their higher-order interactions by using a pipeline developed in house. We demonstrate the predictive power of the density-based regression model and discuss our findings.

## Results

### ChIP-seq data is reproduced and extended

To minimize artifacts, we refined the binding signals of 12 core TFs in mouse ESC publicly available [9] (see *methods*). The ChIP-seq peak datasets generated by various tools are hereafter denoted as FP4_Bowtie, FP4_MAQ, and FP4_Soap2. Also, tag positions mapped by Eland [9] are used for the peak detection (FP4_Eland), and the peak data of Chen et al. is involved (Chen Eland). Thus, we prepared five peak datasets in total.

**Figure 1:**
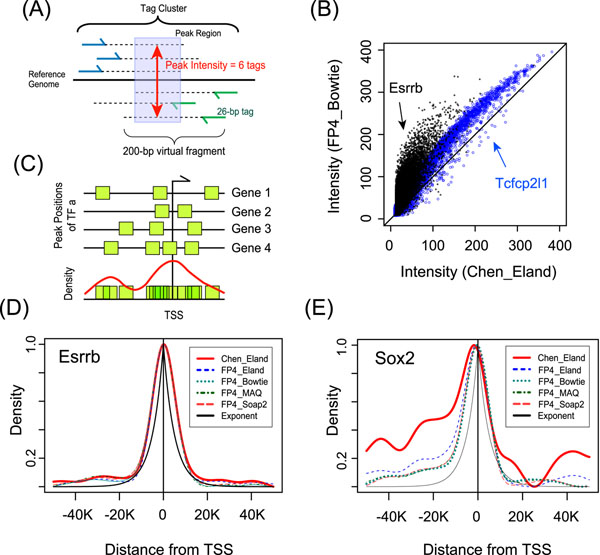
**Schematic representation of ChIP-seq data analysis and examples of TF-binding density profiles.** (A) Definitions of a peak region and its intensity. (B) Example of intensity correlation between the original data and the reanalyzed data. (C) Density estimation from the genome-wide binding locations of a TF. (D)-(E) Example of density profiles in five peak datasets.

Differences in numbers and positions between the remapped data and the original data were investigated. As a result, relatively larger number of uniquely mapped tags and peaks were gained compared to the original data (Table S1-S3 in Additional file 1). In regard to peaks (Table 1), FP4 with the previously mapped tags (FP4_Eland) covers 85-98% of Chen_Eland, and the intensity of overlapped peaks is strongly correlated. Thus, it is deemed that FP4 has reproduced Chen_Eland and extended it with novel peaks in different genomic locations. In contrast, FP4 with remapped tags shows relatively lower reproducibility, whereas peak intensities are still correlated with Chen_Eland except Esrrb (Figure 1B). Similar observations can be found from an independent study [22].

**Table 1:** Reproducibility of newly detected peaks

	Fold Change				Overlap of Chen Eland (%)				Correlation of Peak Intensity			
	Eland	Bowtie	MAQ	Soap2	Eland	Bowtie	MAQ	Soap2	Eland	Bowtie	MAQ	Soap2
c-Myc	1.01	3.26	2.25	3.41	95.12	78.23	77.53	79.78	1.00	0.97	0.98	0.98
E2f1	1.03	1.34	1.36	1.40	85.41	74.67	74.83	75.70	1.00	0.98	0.99	0.99
Esrrb	2.88	3.12	3.29	3.93	99.10	88.62	89.01	90.22	1.00	0.82	0.83	0.83
Klf4	2.30	3.56	3.54	3.83	97.00	91.66	90.90	92.48	1.00	0.94	0.95	0.95
Nanog	1.01	2.15	1.84	2.42	97.93	87.93	90.06	91.69	1.00	0.97	0.99	0.99
n-Myc	1.86	3.24	3.59	3.60	95.39	84.22	85.71	86.15	1.00	0.97	0.97	0.97
Oct4	2.39	6.21	6.72	6.78	96.89	84.26	84.53	87.58	1.00	0.97	0.98	0.98
Smad1	1.49	3.19	3.24	3.53	91.56	75.58	79.84	81.62	1.00	0.85	0.86	0.88
Sox2	1.82	4.23	4.59	4.65	98.37	90.34	90.57	92.93	1.00	0.96	0.98	0.98
Stat3	1.60	8.49	4.80	8.33	97.09	80.99	81.70	84.64	1.00	0.97	0.98	0.98
Tcfcp2l1	1.04	1.73	1.54	1.85	89.05	90.80	91.13	92.73	0.99	0.98	0.99	0.99
Zfx	2.62	3.81	3.94	4.06	94.89	87.67	87.61	88.42	1.00	0.96	0.97	0.97

Fold change is the ratio of newly detected peak number over the original peak number. Overlaps of the original peaks to the newly detected peaks were investigated with 200-bp window. The overlapped peaks were used to calculate the correlation of peak intensity.

The reason why the numbers vary is twofold. First, algorithmic differences in alignment tools cause the different numbers, particularly due to the gapped or ungapped alignment and random indel for mismatches. Second, thresholds for the peak intensity to distinguish experimental noise are different (Table S4 in Additional file 1). That is, Chen et al. used qPCR refinement with small number of peaks, whereas we used Monte Carlo simulation on each chromosome.

### Remapped peaks improve the prediction of gene expression

**Figure 2:**
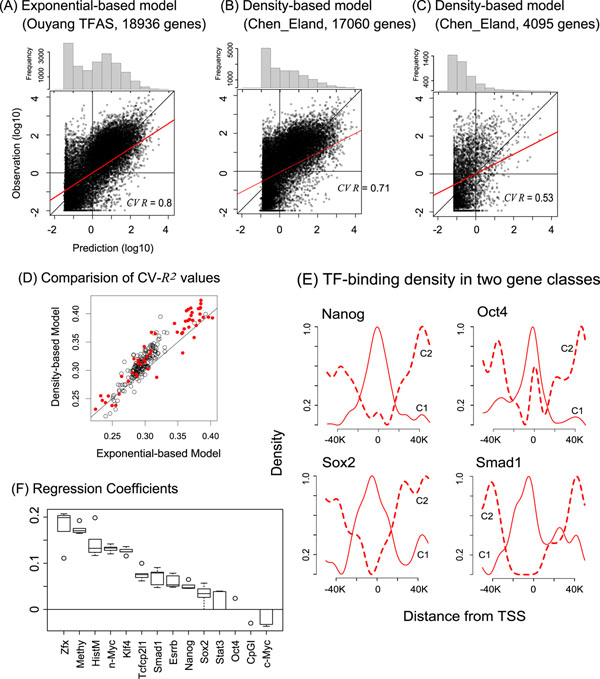
**Predictive results of density-based linear regression model.** (A)-(C) Average correlation coefficient of 10-fold CV in three gene sets. (D) Comparative analysis of two models using ESC-specific gene subsets that co-bound by pluripotent TF pairs. Red solid circles are the cases that the density profiles of a TF pair are significantly different. (E) Distinct binding profiles in two gene classes that are either well explained (C1) or inefficiently explained (C2). (F) Contribution of each TF and epigenetic effects in density-based regression models (Methy: DNA methylation, HistM: histone mark, CpGI: CpG Island).

To assess the importance of TF bindings, Ouyang et al. [21] successfully applied a regression model to the prediction of absolute gene expression in mouse ESC. We first recover this study. Ouyang et al. used TF association strength (TFAS) by summing up peak intensities that are weighted exponentially according to the relative positions from TSSs. We applied the TFAS data to our simple linear regression model shown in equation (1), namely exponential-based regression model. The predictive power of our model is much higher (CV-*R*^2^=0.647) than Ouyang’s model (CV-*R*^2^=0.639), suggesting that the simple regression model is comparable to their PC-regression model (Figure 2A).

Next, we prepared 17060 genes by removing inconsistency between Ouyang’s study and Chen’s study. This procedure is prerequisite for gathering precise TF-binding instances. TFAS data for the genes were calculated by the exactly same procedure of Ouyang et al. As a result, the exponential-based model shows CV-*R*^2^=0.495 with Chen_Eland. In contrast, CV-*R*^2^ increases to 0.542 (FP4_Eland), 0.587 (FP4_Bowtie), 0.581 (FP4_MAQ), and 0.590 (FP4_Soap2).

These results clearly suggest that the proposed simple linear regression model is applicable to the prediction. Furthermore, it has been demonstrated that the peak datasets we remapped give more information for explaining the gene expression.

### Genome-wide locations of TF bindings do not follow exponential distribution

To investigate the characteristics of TF binding sites in ESC, the density profiles of TF-bindings are estimated from each of peak datasets (Figure 1C), then any two density profiles for a TF in different peak datasets are tested by Kolmogorov-Smirnov (KS) test. According to the KS test, the profiles of a TF are almost identical even if the number of mapped tags and peaks are largely different in, say, Esrrb (Figure 1D). The exceptional case is Sox2 in Chen_Eland and FP4_Bowtie (Figure 1E) due in part to the stringent filter used in Chen Eland; e.g. loss of Sox2 peaks in Chen Eland at gene clusters on chromosome X (Figure S1 in Additional file 2).

Importantly, in the same peak dataset, the profiles are significantly different among TFs, e.g. Oct4 and Smad1 in FP4_Bowtie are shown in Figure S2. It is, therefore, thought that spatial preference of TF-bindings cannot be explained by one generalized distribution. In fact, the binding distributions of Nanog, Smad1, Sox2, and Stat3 definitely do not follow the exponential distribution (Figure S2 in Additional file 3).

### Density-based regression model outperforms the exponential-based model

Our observations from the genome-wide distribution of TF binding sites revealed the distinct binding preference from exponential function (Figure 1E). Thus, we use the density profiles as predictors given as equation (2), which we call the density-based regression model. The predictive power of the density-based model with Chen_Eland (Figure 2B) is slightly higher (CV-*R*^2^=0.508) than the exponential-based model (CV-*R*^2^=0.495). Similar results were obtained when other peak datasets were used.

We suspect that the prediction quality of two regression models may depend on downstream genes that cause specific density profiles. To confirm it, we extracted 4095 ESC-specific genes. E2f1 was excluded here due to its excessive regression coefficient [21]. Then, a subset of 4095 genes that is co-bound by a TF pair was prepared. Since the TFs used are well-known essential regulators in ESCs, the TF pairs, such as Oct4 and Sox2, possibly play an important regulatory role in their downstream ESC-specific genes. All subsets by any combination of two TFs have been prepared.

Figure 2D illustrates that the density-based regression model outperforms in many cases. Furthermore, 55 gene subsets that are co-bound by TF pairs whose density profiles are significantly different (*p* < 0.05) were successfully predicted (red solid circles in Figure 2D). These gene subsets cannot be modeled by a generalized exponential function. The results suggest that the spatial preferences of TF bindings are much more dynamically changed in ESC-specific gene subsets rather than observed from all the genes. This is why the density-based model improved the predictive power with respect to the generalized exponential-based model.

### Two gene classes are different in epigenetic patterns

It was demonstrated previously that the absolute gene expression in ESCs is predictable by the ChIP-seq data of core TFs [21]. We also confirmed the high predictive power of the regression model. However, the results strongly rely on certain genes whose ‘*predicted*’ expressions are constantly lower, but ‘*observed*’ expressions are more varied (Figure 2A-C). In Figure 2A, we observed the binomial distribution of predicted expressions that can be partitioned by 1 RPKM (zero on the horizontal axis). We denote C1 for genes where predicted expression is ≥1 RPKM, C2 for the remains. The conspicuous frequency of C2 is also observed from Figure 2B-C. C2 genes in Figure 2C consist of 1205 up- and 1254 down-regulated genes. Further, the subset of C2 (C2′) where observed expression is greater than 1 RPKM consists of 148 up- and 159 down-regulated genes.

**Figure 3:**
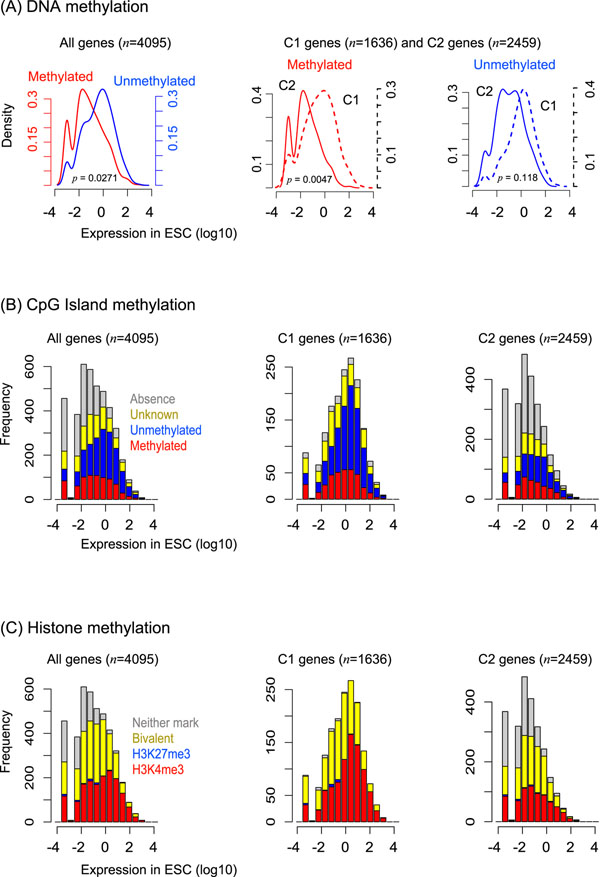
**Epigenetic modifications in ESC-specific genes**. Three epigenetic states observed in genes whose expressions are 4-fold up or down in ESC against EB are considered. Gene class C1 and C2 are well explained and inefficiently explained genes by the regression analysis, respectively.

To characterize the gene classes, we analyzed TF-binding profiles and epigenetic modifications. As a result, in C2 genes, the number of peaks (Figure S3 in Additional file 2) and density profiles (Figure 2E) are apparently different, implying that the small number of TFs bind to distal regions from TSSs. C2 gene promoters are more methylated (Figure 3A). Remarkably, they tend to be absent from CpG islands (Figure 3B), and be marked with neither H2K4me3 nor bivalent domains (Figure 3C). Furthermore, we analyzed gene ontology terms of biological process by DAVID [23]. As a result, C1 was enriched for positive regulation of gene expression (score=8.66), whereas C2 was enriched for neural differentiation (score=34.49). C2′ was enriched for cell morphogenesis (score=2.77).

C2 genes lack the TF-binding instances, implying less direct regulation by the core TFs. This depletion is due in part to excessive non-CpG DNA methylation [16]. Gene ontology analysis shows that C2 genes are often related to differentiation. Thus, they should be preferentially repressed in ESCs. Interestingly, as the histone marks are relatively rare among C2 genes, they are likely to be controlled by other regulatory pathways connecting to the maintenance of self-renewal. One possibility is the competitive binding of additional TFs not involved in this study because of the global open chromatin conformation in ESC [19]. Other possibilities include additional epigenetic patterns and homeostatic regulation, further investigations are required.

### Epigenetic patterns improve the prediction of gene expression

To further understand the epigenetic effects in gene regulation, we add three epigenetic states to the regression models; histone mark (HistM), DNA methylation (Methy), and CpG island (CpGI). Thus, 14 explanatory variables are used. To identify effective variables in the prediction, we reduced the regression model by using the stepwise model selection. Also, 100 runs of computer simulation that randomly assign the epigenetic states were performed.

All models with the epigenetic effects improved CV-*R*^2^ with one to three more variables compared with the models without the epigenetic effects (Table 2). The additional variables are the epigenetic effect terms. The results of simulation support that the improvements are not by the chance. In particular, the density-based models with the epigenetic effects are significantly better when remapped peak datasets are used. Furthermore, overall regression coefficients gathered from all the density-based models in Table 2 show the relative importance of epigenetic effects except CpGI (Figure 2F). Note that the positive-biased activities are consistent with the previous study [24].

**Table 2:** Effects of epigenetic patterns in reduced regression models

Model	Peaks	11 TFs			11TFs + 3 epigenetic effects			Simulation		
		CV-*R*	CV-*R*^2^	Variables	CV-*R*	CV-*R*^2^	Variables	CV-*R*^2^
Exponential	Chen_Eland	0.53	0.282	9	0.58	0.333	12	0.283
	FP4_Eland	0.57	0.319	10	0.59	0.351	12	0.318
	FP4_Bowtie	0.58	0.331	8	0.60	0.356	10	0.330
	FP4_MAQ	0.58	0.335	9	0.60	0.361	12	0.334
	FP4_Soap2	0.58	0.333	9	0.60	0.357	11	0.331

Density	Chen Eland	0.53	0.281	10	0.58	0.334	12	0.282
	FP4_Eland	0.57	0.324	10	0.60	0.358	12	0.325
	FP4_Bowtie	0.59	0.342	9	0.61	0.366	10	0.340
	FP4_MAQ	0.59	0.346	9	0.61	0.370	10	0.345
	FP4_Soap2	0.59	0.344	9	0.61	0.366	12	0.342

### TF interactions wired with epigenetic effects

To investigate the cooperative effects among TFs and epigenetic patterns in gene regulation, we exhaustively searched significant interaction terms from our regression model. First, a subset of ESC-specific genes that are co-bound by a specific TF pair is prepared. Then, the saturated model for the genes is constructed. The model involves 469 variables; 14 main effect terms (11 TFs and 3 epigenetic states) and 455 higher-order interaction terms (all the possible pairwise and triplewise interactions). Finally, our pipeline greedily identifies important variables (see *methods*). This procedure is independently performed with each of five peak datasets.

**Figure 4:**
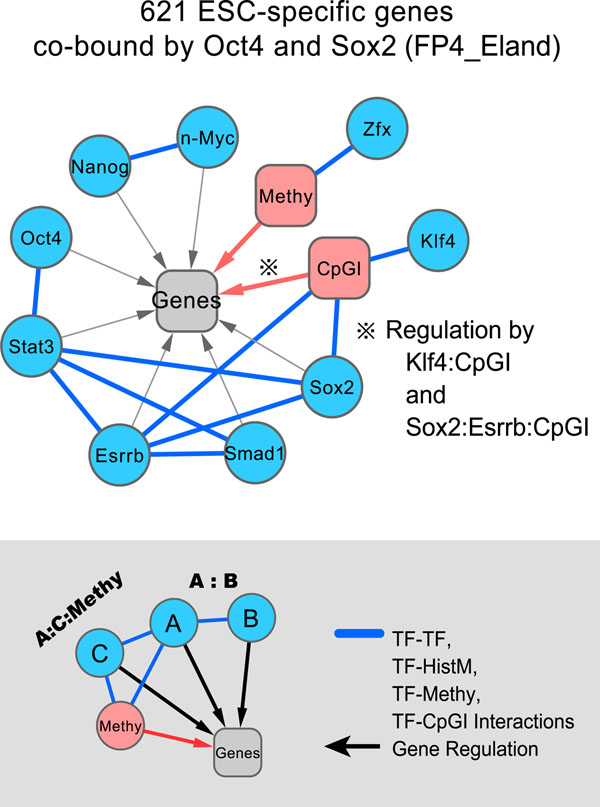
**Example of regulatory network of TF interactions with epigenetic effects**. This network was generated by the connectivity of nodes in all interaction terms. For example, an interaction term A:B:C is split into three interactions, A:B, A:C, and B:C. Then, the nodes are linked to each other and to the target gene set. A pairwise interaction with an epigenetic effect is treated differently. For example, in the case of B:Methy, B is not linked to the target gene set.

In total, 215 models were identified in which the predictive power is higher than the models without higher-order terms. These models contained 6-30 variables including at least one interactive term. As an example, the regression model for genes co-bound by Oct4 and Sox2, a well-known pluripotent complex [9,25], contained 15 terms and improved CV-*R*^2^=0.4126 from 0.3837 in the model with only 14 main effect terms. This model suggests that 7 interactive terms are important in the explanation of target gene expression. Among them, 3 terms are mediated by the epigenetic effects. The network representation of this model highlights the importance of signaling receptors (Stat3 and Smad1), activating Oct4/Sox2 complex [9] as well as Klf4/CpGI [26], and the interaction of Zfx/Methy newly found here (Figure 4).

With considering the redundancy and conservativity, we represented the interactive terms of 215 models as a network (Figure S4 in Additional file 2). As a result, 19 gene sets covering approximately 86% of genes (3523 out of 4095 genes) were linked by 28 regulatory edges of the epigenetic effects that are commonly found in the five peak datasets (Figure S4 in Additional file 2). These results suggest that the cooperative interactions between TF and the epigenetic state are indispensable to explain the majority of gene expression in ESCs. In addition, we confirmed that the regression coefficients in Figure 2F are dramatically changed in the regression of given gene sets, and also CpGI significantly contributes to the prediction of gene expression (Figure 4).

## Discussion

ESCs are the widely accepted source for the study of many biological principles. Despite recent advances in our understanding of biological systems, the gene regulation in ESCs is only incompletely understood. To explore the regulatory mechanism underlying in ESCs, we constructed a predictive model for explaining the absolute gene expression in mouse ESC. This model uses a novel density-based approach to exploit the recent massive parallel sequencing straightforwardly.

We first reanalyzed the publicly available ChIP-seq data for 12 well-known pluripotent core TFs [9], and retrieved the reproduced and extended TF-binding sites and intensities (Table 1). Using our regression model based on the exponential function [21], we found that the remapped peaks are more informative to explain the gene expression (Table 2). Therefore, we concluded that the algorithmic differences in computer tools for ChIP-seq data significantly affect the downstream analysis. Analyzing the heterogeneous peak datasets in a comparative manner, we found that the spatial binding preference of each TF is well conserved in all the datasets, whereas the preferences of TFs in a dataset are significantly different from each other (Figure 1D-E). These results imply that density profiles are better explanatory variables than the generalized exponential function. In fact, the predictive power of density-based model is constantly higher than the exponential-based model (Figure 2A-C, Table 2). Even if the density profiles are dynamically changed in certain downstream genes, the proposed model is still outstanding (Figure 2D).

Unexpectedly, we found two gene classes that are either well explained or inefficiently explained by the regression model. The latter class genes have less binding instances of the pluripotent TFs (Figure 2E), possibly related to excessive DNA methylation (Figure 3A). The gene classes show apparently different characteristics in epigenetic modifications (Figure 3), suggesting that they are likely to be under control in different regulatory mechanisms. In the present study, we simply combined the discrete epigenetic states with the powerful density-based model. This model significantly improved the predictive power (Table 2). Investigating higher-order interactions among the predictors, we found that the cooperative interactions between TF and epigenetic pattern are indispensable for regulating approximately 86% of ESC-specific genes (Figure S4 in Additional file 2). These results suggest that the relative importance of epigenetic effects to regulate the gene expression in ESCs, supporting the general idea [14,15].

We proposed a powerful regression model, and uncovered the relative importance of epigenetic regulation in ESCs. Overall prediction quality is still insufficient. As future works, comprehensive representation of epigenetic patterns is required, and additional or alternative TFs in ESCs should be considered.

## Methods

### Data acquisition

#### ChIP-seq data and gene expression

Raw tag sequences and a control library were downloaded from GEO database (GSE11431). High-quality 26 base pair (bp) tags that have less than three ambiguous bases were mapped to mm8 by Bowtie [27], MAQ [28], and Soap2 [29] with allowing two mismatches. Only uniquely mapped tags were extended to 200-bp virtual fragments (Figure 1A). FP4 (FindPeaks 4.0) [30] detected significant peak regions. Monte Carlo simulation was performed on each chromosome to calculate false discovery rate (FDR). Also, the fold enrichment of tags in each peak region over remapped control tags was measured. Finally, we prepared peaks by criteria, FDR <5% and 5-fold enrichment.

For the absolute gene expression, the number of tags per kilobase of exon region per million mapped tags (RPKM) [18] for 18936 mouse genes in ESC and in embryoid body (EB) were prepared from [21]. Positional information of transcription start sites (TSSs) of 17443 Refseq genes in mm8 were prepared from [9]. Removing inconsistent gene IDs between RPKM data and TSS data, we compiled 17060 genes. We prepared 4095 ESC-specific genes whose expressions are 4-fold up- or down-regulated in ESC over EB. The dataset used in here is available at http://www.hgc.jp/~park/research/.

#### Epigenetic modifications

DNA methylation maps are prepared from two datasets that use different high-throughput detection methods [16,17]. Methylation states of high-CpG-density promoters (GC content ≥0.55) are defined by mean methylation levels; unmethylated if mean ≤0.25, methylated if mean ≥0.75. The genome-wide distribution of CpG islands and histone mark were downloaded from UCSC genome browser. We consider three histone states; histone H3 lysine 4 trimethylation (H3K4me3), an active mark of expression, H3 lysine 27 trimethylation (H3K27me3), a repressive mark, and bivalent domain of H3K4me3 and H3K27me3, a ‘poised’ mark of expression.

### Estimation of TF binding density

Given a genome-wide location map of a TF-bindings, all peak positions were converted to relative positions to the nearest TSSs. Gaussian kernel density function (bandwidth=300 bps) estimated the density profile of the TF-bindings within ±50K bps. The profile was normalized into range of [0, 1] by dividing by the maximal density height.

### Regression model

We use a multivariate regression model(1)

where *Y_i_* is the expression of gene *i*, *S_ij_* is the score of the *j*th TF on gene *i*, *w_j_* is the regression coefficient of the *j*th TF, and *e_i_* is the error term. The score *S_ij_* is given by(2)

where *g_k_* is the perk intensity of the *k*th binding peak of the *j*th TF, *F_j_* is the normalized density function for the *j*th TF, and *l_k_* is the relative position of the *k*th peak to TSS of gene *i*. Note that a small value is added to *Y_i_* for the logarithm.

### Adding epigenetic effects

Discrete values representing epigenetic states of a gene *i* are added to the regression model(3)

where *H* is the type of histone mark (neither mark=1.0, H3K27me3=2.0, bivalent mark=3.0, H3K4me3=4.0), *M* is the DNA methylation (no annotation=1.0, methylation=2.0, unmethylation=3.0), *C* is the CpG island (absence=1.0, presence=2.0), and *α*, *β*, *γ* are the regression coefficients for *H*, *M*, *C*, respectively.

### Fitting and reducing regression models

Explanatory variables in a regression model are log-transformed and quantile-normalized. 10 runs of 10-fold cross validation (CV) measure the average correlation coefficient (CV-*R*) and the average proportion of variation explained by the model (CV-*R*^2^). The stepwise model selection is done by stepAIC in R language with the backward and forward procedure. The regression model with higher-order interactions are reduced by a pipeline developed in house; ANOVA in R language first diagnoses the significance of each explanatory variable in the given saturated model. Next, significant variables (*p* < 0.05 in F-test) are gathered. Finally, the best model is constructed by adding and removing the collected variables one by one in increasing order of *p*-value until CV-*R*^2^ is not improved anymore.

## Competing interests

The authors declare that they have no competing interests.

## Supplementary Material

Additional file 1 **Extended analysis of ChIP-seq data** This file provides tables including the summary of tag mapping (Table S1), the fold change of remapped tags over the original data (Table S2), the number of peaks in five datasets (Table S3), and the thresholds used to detect significant peaks (Table S4). Click here for file

Additional file 2 **Comprehensive analysis of gene regulation in mouse ESC** This file provides figures including an example of peak distributions (Figure S1), TF-binding instances in two gene classes (Figure S3), and the regulatory network of TFs wired with epigenetic effects (Figure S4). Click here for file

Additional file 3 **Density profile of 12 TFs** This file provides the density profiles of 12 core TFs in five peak datasets (Figure S2). Click here for file
