# Supplementary material for: A regression analysis of gene expression in ES cells reveals two gene classes that are significantly different in epigenetic patterns
Source: BMC Bioinformatics. 2011 Feb 15;12(Suppl 1):S50. doi: 10.1186/1471-2105-12-S1-S50 (PMC3044308; doi:10.1186/1471-2105-12-S1-S50)
Supplement: Additional file 1 — Extended analysis of ChIP-seq data This file provides tables including the summary of tag mapping (Table S1), the fold change of remapped tags over the original data (Table S2), the number of peaks in five datasets (Table S3), and the thresholds used to detect significant peaks (Table S4). [file 1471-2105-12-S1-S50-S1.pdf]

Table S1

	Tags	Uniquely mapped tags				Mappability (%)		
		Eland	Bowtie	MAQ	Soap2	Bowtie	MAQ	Soap2
c-Myc	26,507,905	6,637,404	10,706,401	11,570,815	12,359,761	63.26	62.10	63.48
E2f1	29,877,423	6,026,155	10,354,841	10,938,919	11,680,246	52.18	51.14	52.53
Esrrb	25,284,321	3,609,843	11,831,415	12,698,806	13,351,007	65.92	65.12	66.23
Klf4	19,658,061	3,807,970	6,688,461	7,105,939	7,573,812	51.42	50.89	52.63
Nanog	23,210,244	8,424,102	10,228,002	10,984,575	11,775,466	68.89	67.52	69.93
n-Myc	22,818,575	4,823,212	7,711,070	8,412,032	8,787,237	51.80	51.94	52.84
Oct4	23,339,676	4,911,144	8,051,461	8,718,505	9,145,158	53.45	53.46	54.46
Smad1	19,348,766	3,338,896	5,283,929	5,261,880	6,140,352	45.09	41.07	45.33
Sox2	22,221,844	4,821,446	8,271,344	9,004,099	9,383,822	56.12	56.51	57.39
Stat3	21,575,655	5,351,116	8,788,899	9,668,243	10,083,166	66.70	66.82	67.64
Tcfcp2l1	30,624,944	8,787,961	11,073,220	11,685,125	12,476,979	52.45	51.40	52.77
Zfx	17,734,559	3,844,429	7,406,375	7,781,303	8,372,722	62.51	60.69	62.74

Table S2

	Fold Change				Overlap (%)			
	Eland	Bowtie	MAQ	Soap2	Eland	Bowtie	MAQ	Soap2
c-Myc	1.00	1.61	1.74	1.86	100.00	90.30	96.84	99.42
E2f1	1.00	1.72	1.82	1.94	100.00	92.90	97.28	99.96
Esrrb	1.00	3.28	3.52	3.70	100.00	67.31	68.88	69.78
Klf4	1.00	1.76	1.87	1.99	100.00	90.37	94.49	97.31
Nanog	1.00	1.21	1.30	1.40	100.00	90.56	96.22	98.46
n-Myc	1.00	1.60	1.74	1.82	100.00	90.11	96.22	98.12
Oct4	1.00	1.64	1.78	1.86	100.00	90.99	96.08	98.43
Smad1	1.00	1.58	1.58	1.84	100.00	86.89	90.71	97.43
Sox2	1.00	1.72	1.87	1.95	100.00	90.91	96.07	98.26
Stat3	1.00	1.64	1.81	1.88	100.00	91.10	97.44	99.22
Tcfcp2l1	1.00	1.26	1.33	1.42	100.00	83.79	87.50	89.38
Zfx	1.00	1.93	2.02	2.18	100.00	91.41	96.50	99.99

- Table S1
  - Mapping result of high-quality 26-bp tags derived from ChIP-seq data (GSE11431). Eland has been done by Chen et. al. (Cell 2008, 133:1106-1117). Mappability is calculated by (unique hits + multiple hits) / total 26-bp tags.
- Table S2
  - Fold change of the number of mapped tags over that of Chen's data. Overlaps have been checked by that at least one base of 26-bp tags of Chen_Eland overlaps to any of tags mapped in this study.

Table S3

	Chen	FindPeaks				Overlap of Chen's peaks			
	Eland	Eland	Bowtie	MAQ	Soap2	Eland	Bowtie	MAQ	Soap2
c−Myc	3422	3441	11150	7708	11665	3255	2677	2653	2730
E2f1	20699	21378	27834	28226	29036	17680	15455	15490	15670
Esrrb	21647	62309	67634	71211	84965	21452	19183	19267	19529
Klf4	10875	24988	38763	38449	41619	10549	9968	9885	10057
Nanog	10343	10432	22219	18992	25005	10129	9095	9315	9483
n−Myc	7182	13348	23291	25813	25852	6851	6049	6156	6187
Oct4	3761	9000	23349	25281	25505	3644	3169	3179	3294
Smad1	1126	1683	3596	3643	3978	1031	851	899	919
Sox2	4526	8257	19127	20764	21062	4452	4089	4099	4206
Stat3	2546	4079	21614	12231	21211	2472	2062	2080	2155
Tcfcp2l1	26910	28015	46466	41402	49798	23963	24433	24523	24953
Zfx	10338	27085	39361	40738	41935	9810	9063	9057	9141

Table S4

	Chen	FP4 (chr1, chr2, chr3, ..., chrX)	
		Eland	Bowtie
c−Myc	7	7, 7, 7, 7, 7, 8, 7, 8, 8, 7, 8, 7, 8, 7, 8, 7, 7, 7, 7, 6	8, 8, 8, 8, 8, 8, 8, 8, 9, 8, 9, 8, 8, 8, 8, 8, 8, 8, 8, 6
E2f1	6	6, 6, 6, 6, 6, 6, 6, 6, 6, 6, 6, 6, 6, 6, 6, 6, 6, 6, 6, 5	7, 7, 7, 7, 7, 7, 7, 7, 7, 7, 7, 7, 7, 7, 7, 7, 7, 7, 7, 5
Esrrb	5	5, 5, 5, 5, 5, 5, 5, 5, 5, 5, 6, 5, 5, 5, 5, 5, 5, 5, 5, 5	6, 6, 6, 6, 6, 6, 6, 6, 6, 6, 7, 6, 6, 6, 6, 6, 7, 6, 6, 5
Klf4	5	6, 6, 6, 6, 6, 6, 6, 6, 6, 6, 6, 6, 6, 6, 6, 6, 6, 6, 6, 5	6, 6, 6, 6, 6, 6, 6, 6, 6, 6, 6, 6, 6, 6, 6, 6, 6, 6, 6, 5
Nanog	7	8, 7, 7, 7, 7, 7, 7, 8, 8, 7, 8, 7, 7, 7, 8, 7, 7, 7, 8, 6	8, 8, 8, 8, 8, 8, 8, 8, 8, 8, 8, 8, 8, 8, 8, 8, 8, 8, 8, 6
n−Myc	6	6, 6, 6, 6, 7, 6, 6, 7, 6, 6, 7, 6, 6, 6, 6, 6, 6, 6, 6, 5	7, 7, 7, 7, 7, 7, 7, 7, 7, 7, 7, 7, 7, 7, 7, 7, 7, 7, 7, 5
Oct4	6	7, 7, 6, 7, 7, 6, 7, 7, 7, 6, 7, 6, 7, 7, 7, 7, 7, 6, 7, 5	7, 7, 7, 7, 7, 7, 7, 7, 7, 7, 7, 7, 7, 7, 7, 7, 7, 7, 7, 6
Smad1	6	7, 6, 7, 6, 6, 6, 6, 7, 6, 6, 6, 6, 7, 6, 7, 6, 7, 7, 6, 5	8, 8, 7, 7, 7, 7, 7, 7, 7, 7, 7, 7, 7, 7, 7, 8, 7, 7, 7, 6
Sox2	6	7, 7, 6, 7, 7, 6, 7, 7, 7, 6, 7, 7, 6, 6, 7, 6, 7, 7, 7, 5	7, 7, 7, 7, 7, 7, 7, 7, 7, 7, 7, 7, 7, 7, 7, 7, 7, 7, 7, 6
Stat3	6	7, 7, 7, 7, 7, 7, 7, 7, 7, 7, 7, 7, 7, 7, 7, 7, 7, 7, 7, 6	8, 8, 7, 7, 7, 7, 7, 8, 7, 7, 8, 7, 7, 7, 7, 7, 7, 8, 7, 6
Tcfcp2l1	6	7, 7, 6, 7, 7, 7, 7, 7, 7, 7, 7, 7, 7, 7, 7, 7, 7, 7, 7, 5	7, 7, 7, 7, 7, 7, 7, 7, 7, 7, 7, 7, 7, 7, 7, 7, 7, 7, 7, 5
Zfx	5	6, 6, 6, 6, 6, 6, 6, 6, 6, 6, 6, 6, 6, 6, 6, 6, 6, 6, 6, 5	6, 6, 6, 6, 6, 6, 6, 6, 6, 6, 6, 6, 6, 6, 6, 6, 6, 6, 6, 5

	Chen	FP4 (chr1, chr2, chr3, ..., chrX)	
		MAQ	Soap2
c−Myc	7	8, 9, 8, 8, 9, 8, 8, 9, 9, 9, 9, 8, 9, 8, 8, 8, 8, 9, 8, 6	9, 9, 8, 8, 9, 9, 8, 9, 9, 9, 9, 9, 9, 8, 8, 9, 8, 9, 9, 6
E2f1	6	7, 7, 7, 7, 7, 7, 7, 7, 7, 7, 7, 7, 7, 7, 7, 7, 7, 7, 7, 5	7, 7, 7, 7, 7, 7, 7, 7, 7, 7, 7, 7, 7, 7, 7, 7, 7, 7, 7, 6
Esrrb	5	6, 6, 6, 6, 7, 6, 6, 7, 7, 6, 7, 6, 6, 6, 7, 6, 7, 6, 7, 5	6, 7, 6, 7, 7, 6, 7, 7, 7, 7, 7, 6, 6, 6, 6, 6, 7, 6, 7, 5
Klf4	5	6, 6, 6, 6, 6, 6, 6, 6, 6, 6, 7, 6, 6, 6, 6, 6, 6, 6, 6, 5	6, 6, 6, 6, 6, 6, 6, 7, 6, 6, 7, 6, 6, 6, 6, 6, 6, 6, 6, 5
Nanog	7	8, 8, 8, 8, 8, 8, 8, 8, 8, 8, 8, 8, 8, 8, 8, 8, 8, 8, 8, 6	8, 8, 8, 8, 8, 8, 8, 9, 8, 8, 9, 8, 8, 8, 8, 8, 8, 8, 8, 6
n−Myc	6	7, 7, 7, 7, 7, 7, 7, 7, 7, 7, 7, 7, 7, 7, 7, 7, 7, 7, 7, 5	7, 7, 7, 7, 7, 7, 7, 7, 7, 7, 7, 7, 7, 7, 7, 7, 7, 7, 7, 6
Oct4	6	7, 7, 7, 7, 7, 7, 7, 7, 7, 7, 8, 7, 7, 7, 7, 7, 7, 7, 7, 6	7, 7, 7, 7, 7, 7, 7, 8, 8, 7, 8, 7, 7, 7, 7, 7, 7, 7, 8, 6
Smad1	6	7, 7, 7, 7, 7, 7, 7, 8, 7, 8, 7, 7, 7, 7, 8, 7, 7, 8, 7, 6	8, 8, 8, 7, 7, 7, 7, 8, 8, 8, 8, 8, 8, 7, 8, 8, 7, 8, 8, 6
Sox2	6	7, 7, 7, 7, 7, 7, 7, 8, 7, 7, 8, 7, 7, 7, 7, 7, 7, 7, 7, 6	7, 7, 7, 7, 7, 7, 7, 8, 8, 7, 8, 7, 7, 7, 7, 7, 7, 7, 8, 6
Stat3	6	8, 8, 8, 7, 8, 7, 7, 8, 8, 8, 8, 7, 8, 8, 8, 8, 7, 8, 8, 6	8, 8, 8, 8, 8, 8, 8, 8, 8, 8, 8, 8, 8, 8, 8, 8, 8, 8, 8, 6
Tcfcp2l1	6	7, 7, 7, 7, 7, 7, 7, 7, 7, 7, 7, 7, 7, 7, 7, 7, 7, 7, 7, 5	7, 7, 7, 7, 7, 7, 7, 7, 7, 7, 7, 7, 7, 7, 7, 7, 7, 7, 7, 6
Zfx	5	6, 6, 6, 6, 6, 6, 6, 6, 6, 6, 7, 6, 6, 6, 6, 6, 6, 6, 6, 5	6, 6, 6, 6, 6, 6, 6, 7, 6, 6, 7, 6, 6, 6, 6, 6, 6, 6, 6, 5

- Table S3
  - Number of peaks. FP4 (FindPeaks 4.0) detected tag enrichments. Overlaps of Chen_Eland to ours have been checked by whether a Chen's peak center co-localizes with any of peak centers detected by FP4 within 200-bp window.
- Table S4
  - Thresholds used to detect significant peaks (FDR < 5). Chen et al. have determined thresholds by qPCR. We performed Monte Carlo simulations on each chromosome.
