# Supplementary material for: A regression analysis of gene expression in ES cells reveals two gene classes that are significantly different in epigenetic patterns
Source: BMC Bioinformatics. 2011 Feb 15;12(Suppl 1):S50. doi: 10.1186/1471-2105-12-S1-S50 (PMC3044308; doi:10.1186/1471-2105-12-S1-S50)
Supplement: Additional file 2 — Comprehensive analysis of gene regulation in mouse ESC This file provides figures including an example of peak distributions (Figure S1), TF-binding instances in two gene classes (Figure S3), and the regulatory network of TFs wired with epigenetic effects (Figure S4). [file 1471-2105-12-S1-S50-S2.pdf]

Figure S1

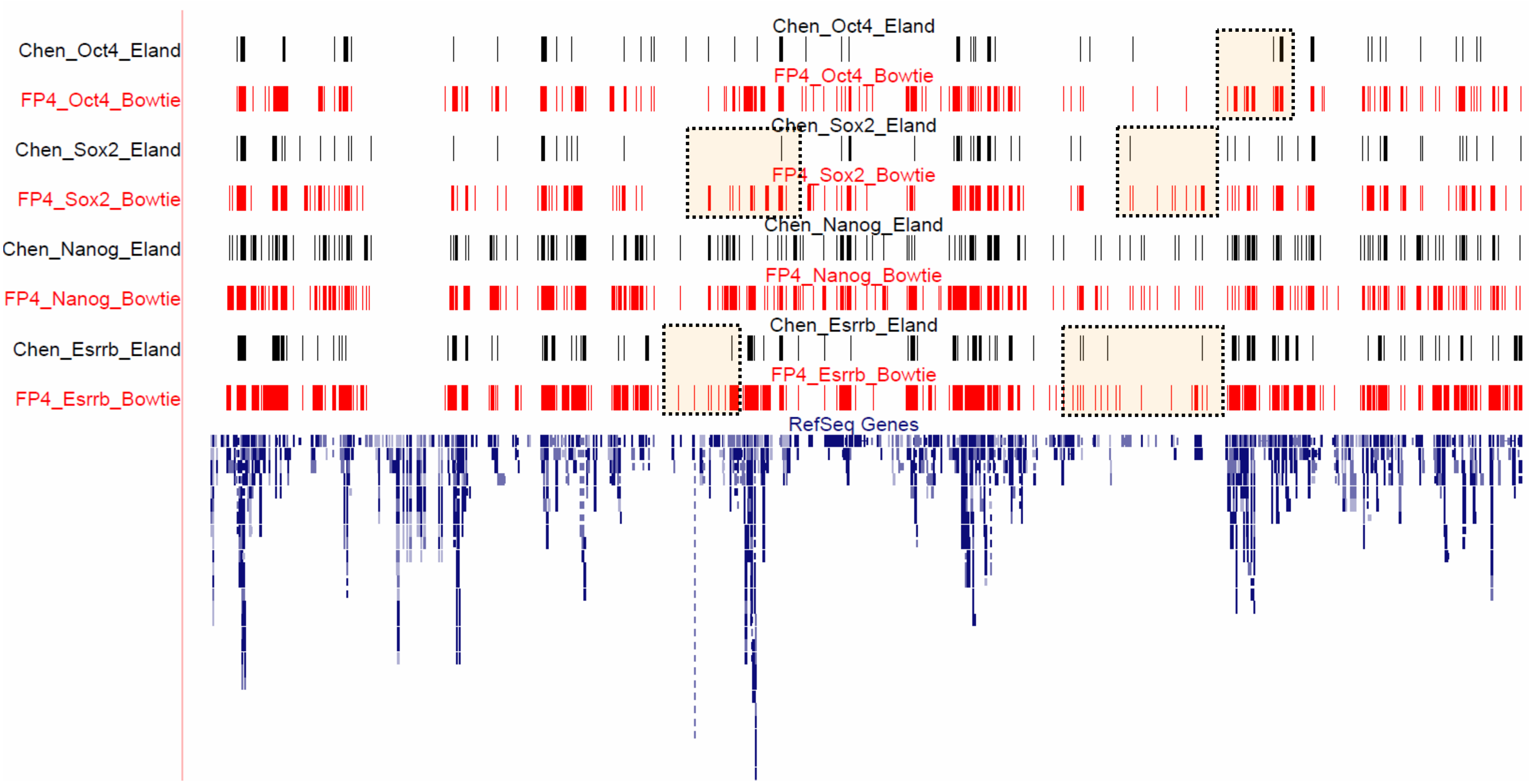

- Figure S1
  - Example of the peak distributions of Oct4, Sox2, Nanog, and Esrrb on chromosome X in mm8. The bottom track shows positions of Refseq genes. Because loss of peaks at gene clusters is observed (e.g. dotted boxes), it is implied that relatively stringent thresholds have been used in Chen_Eland, especially for Oct4, Sox2, and Esrrb.

Figure S3

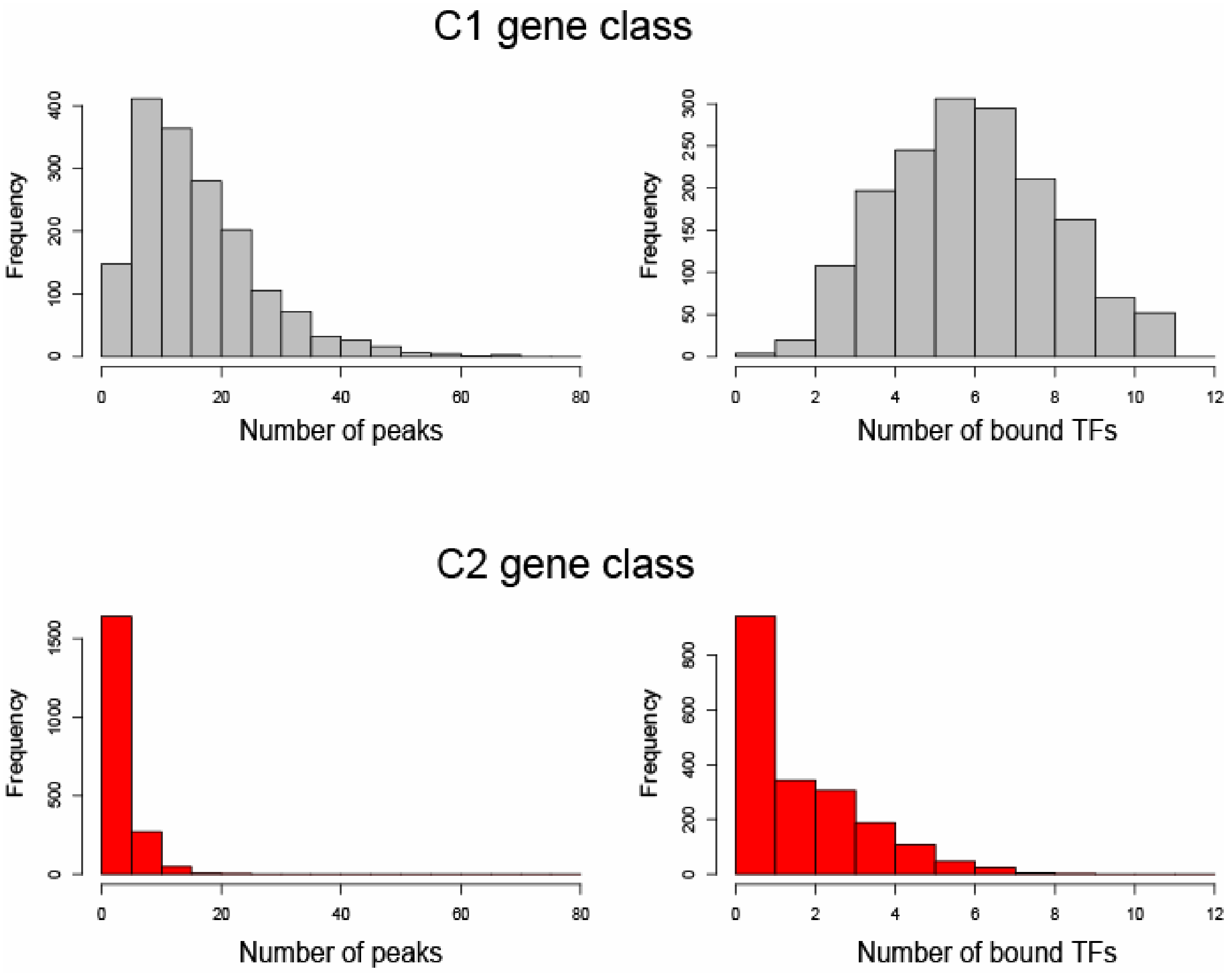

- Figure S3
  - Distribution of TF-bindings in two ESC-specific gene classes: C1 for genes in >= 1 RPKM, C2 for genes in < 1RPKM. TF-bindings are depleted in C2 class.

Figure S4

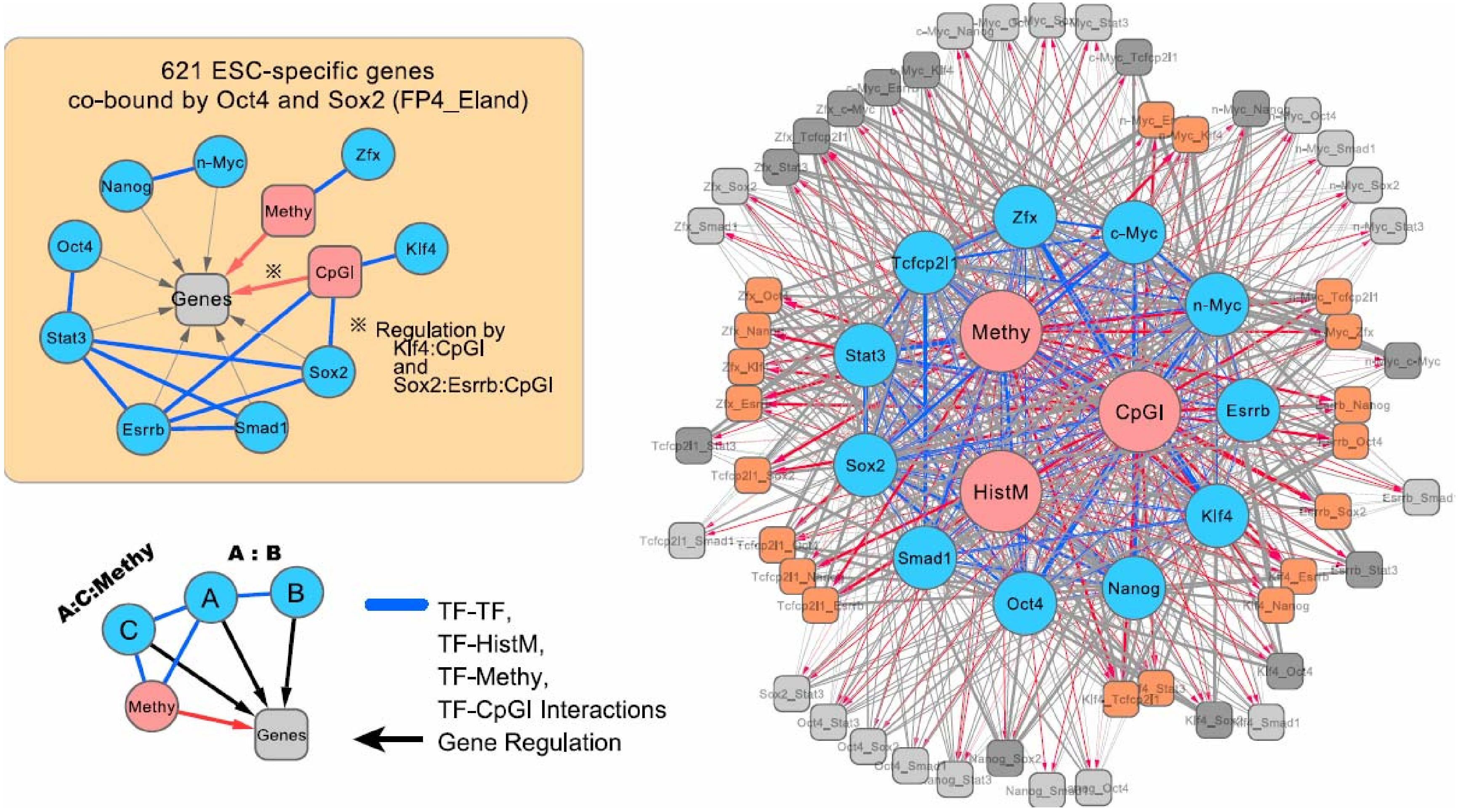

- Figure S4: Regulatory network of transcription factor interactions with epigenetic effects in ES cell
  - The network provides 140 edges (blue lines) representing higher-order interactions in 52 downstream gene sets that co-bound by a specific TF pair (rectangles). 670 edges (grey and red lines) represent the regulatory effects from the interactions to 52 gene sets. Among them, 128 edges are from the epigenetic effects (red lines). 149 of 670 edges connecting to 32 gene sets are commonly found in all the five peak datasets (thicker grey and thicker red lines), and 19% edges (28/149) are the epigenetic effects (thicker red lines).
