# Supplementary figures and images for: A regression analysis of gene expression in ES cells reveals two gene classes that are significantly different in epigenetic patterns
Source: BMC Bioinformatics. 2011 Feb 15;12(Suppl 1):S50. doi: 10.1186/1471-2105-12-S1-S50 (PMC3044308; doi:10.1186/1471-2105-12-S1-S50)

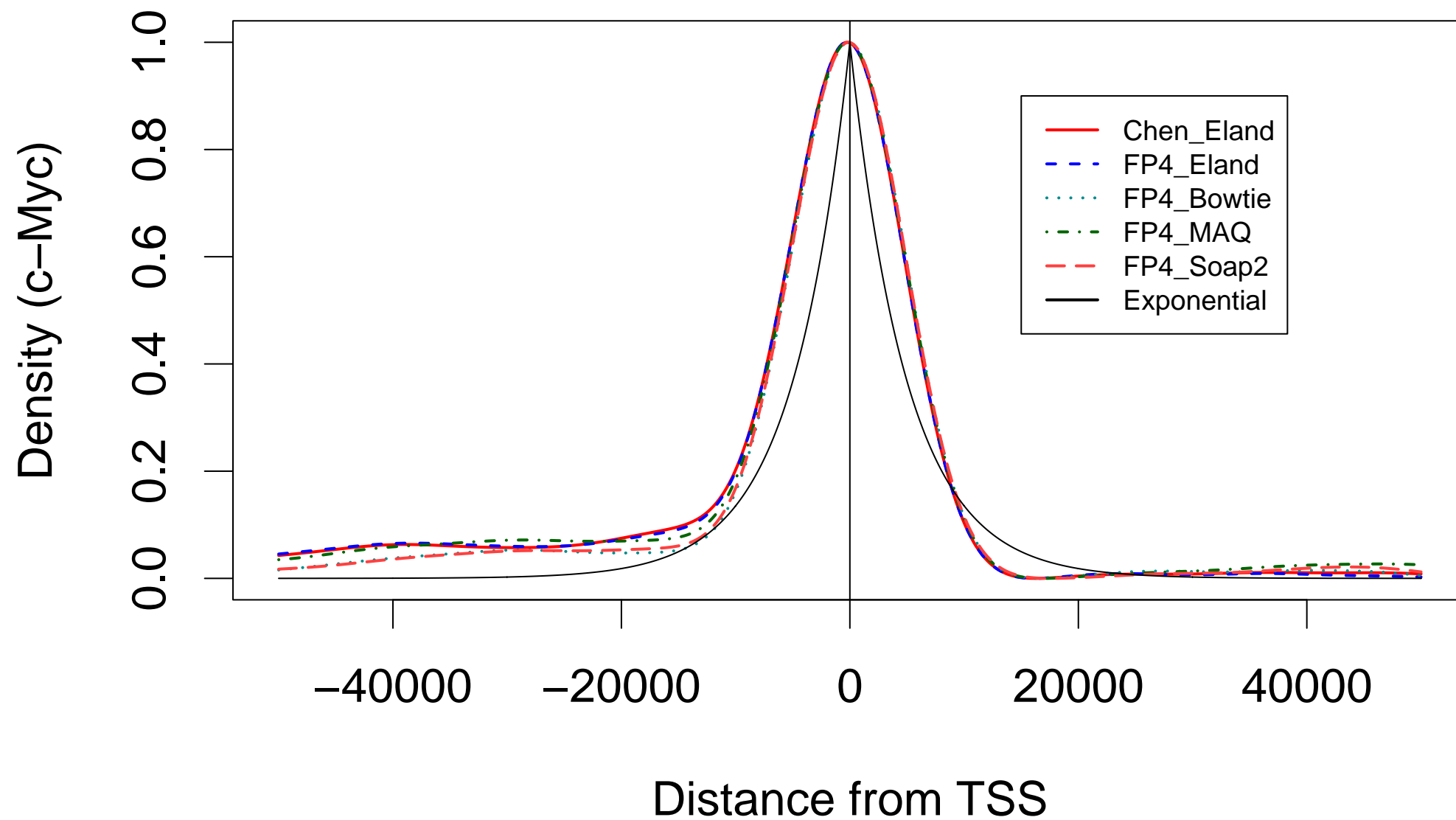

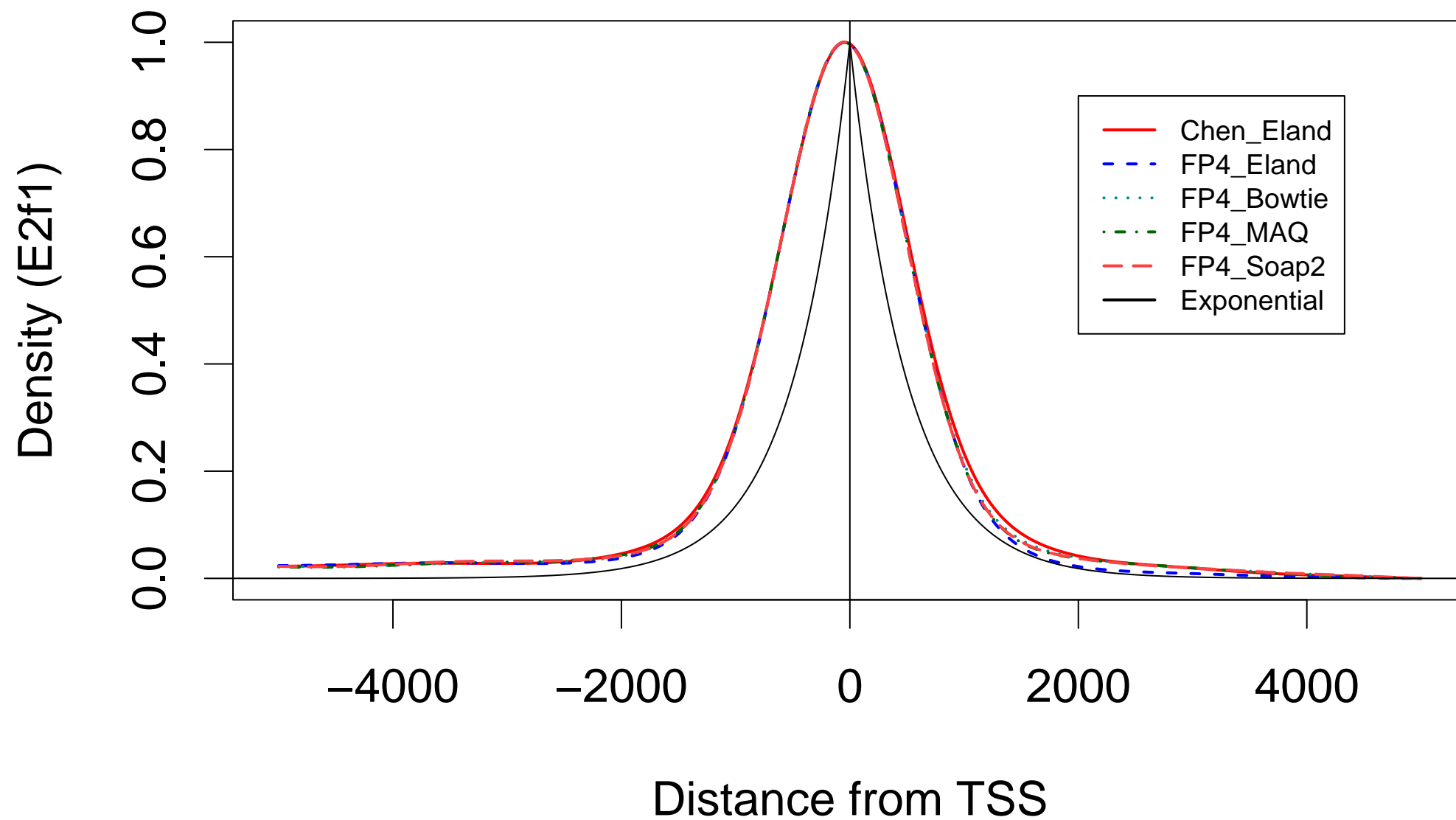

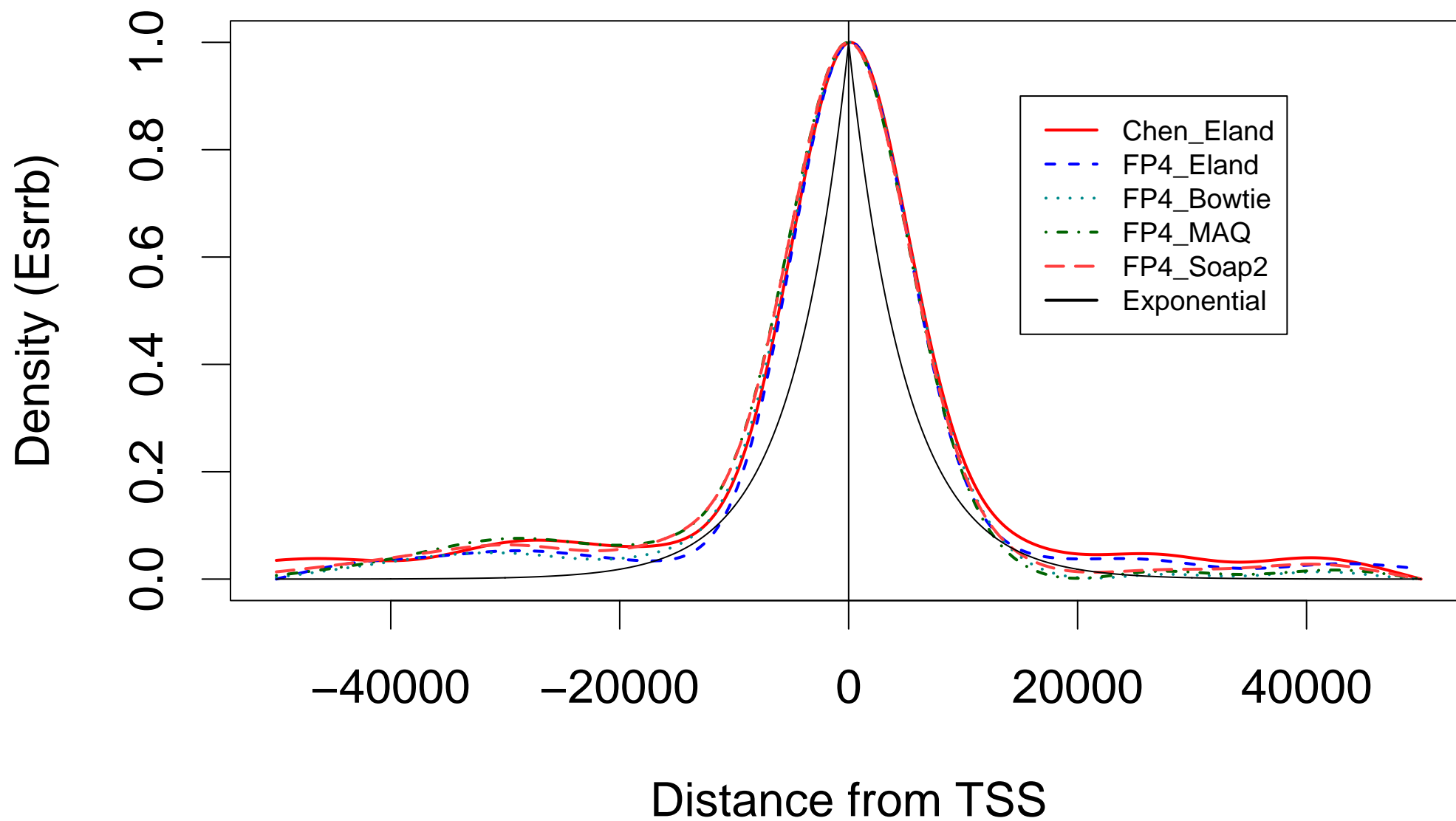

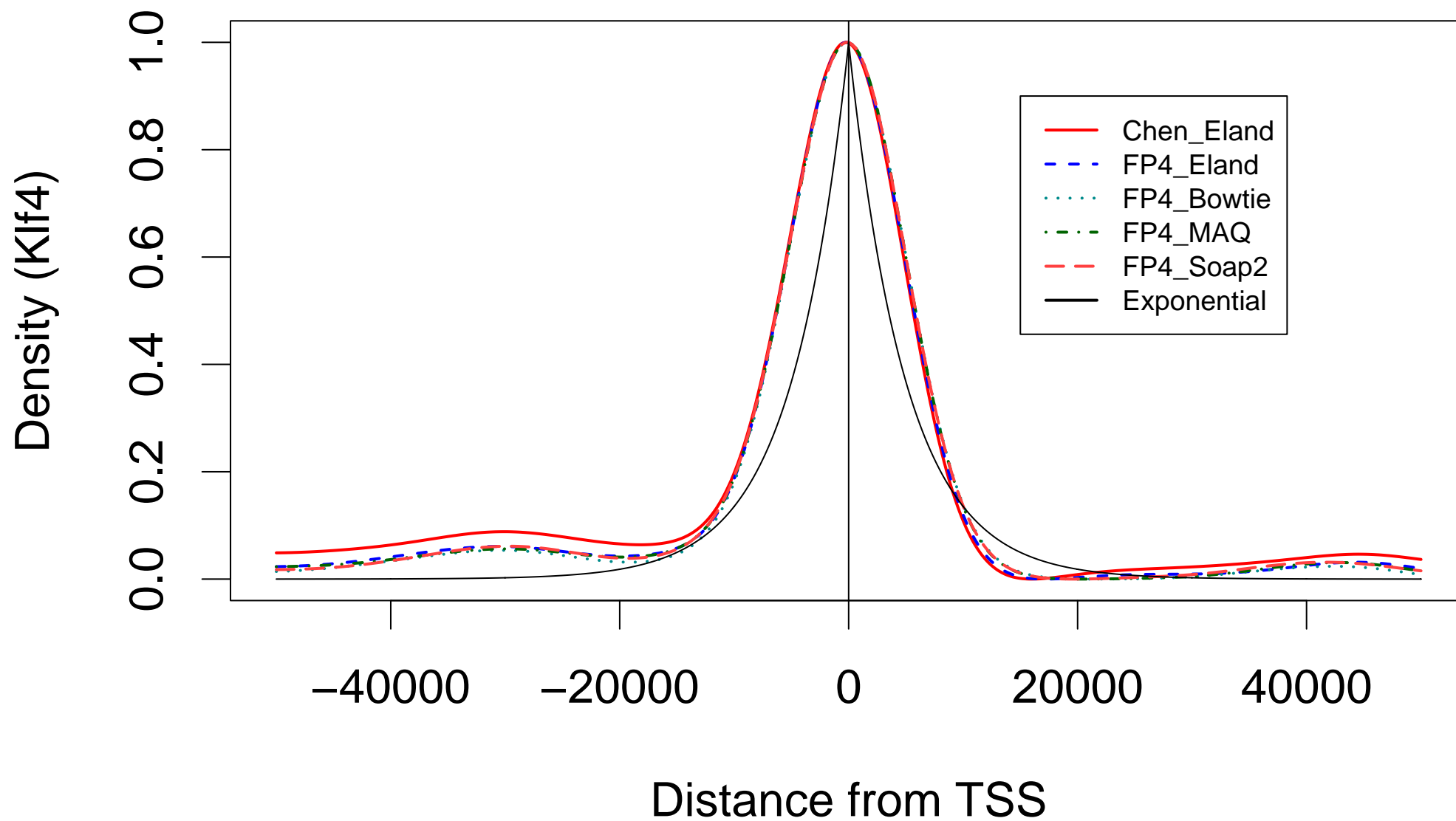

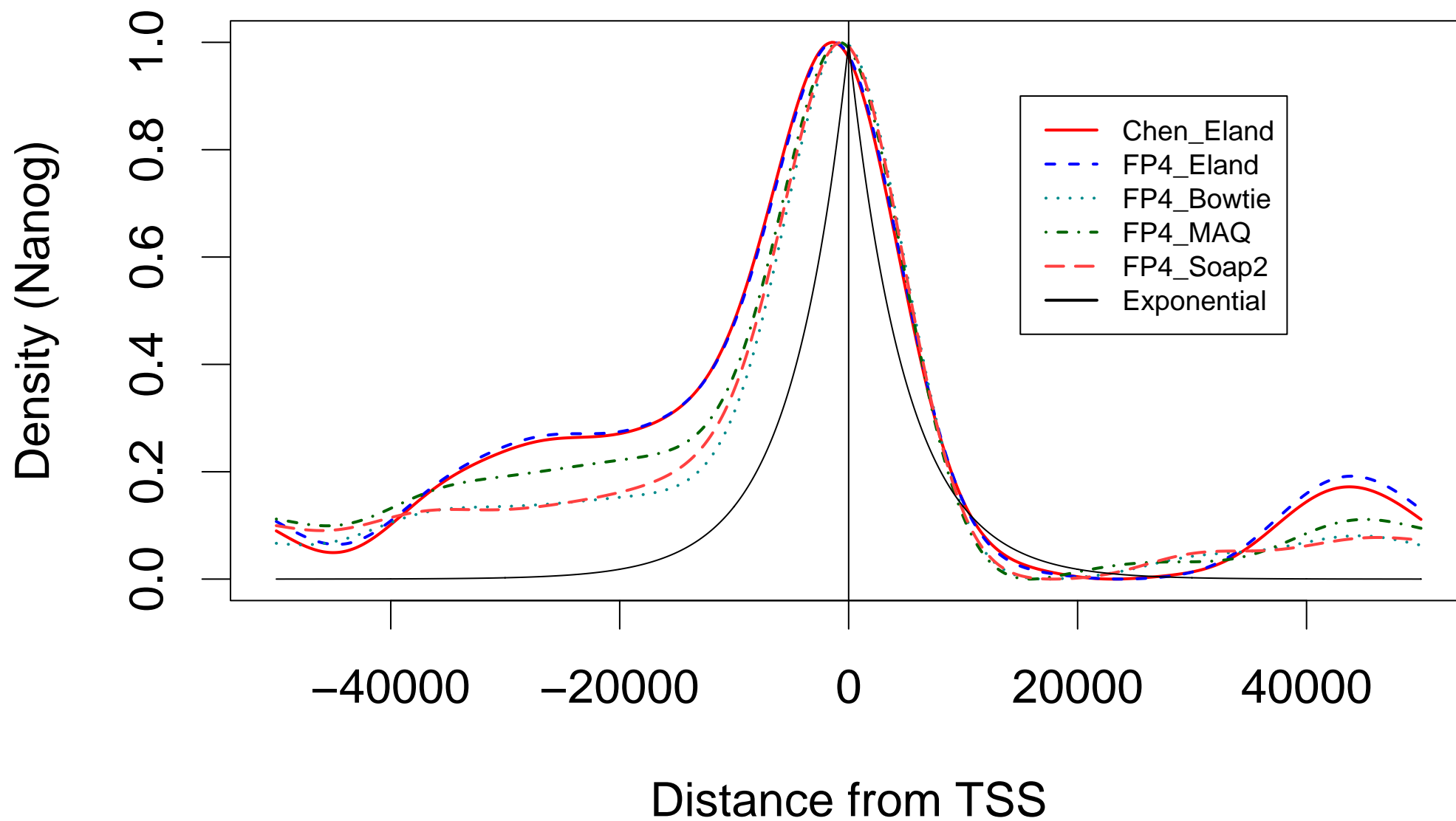

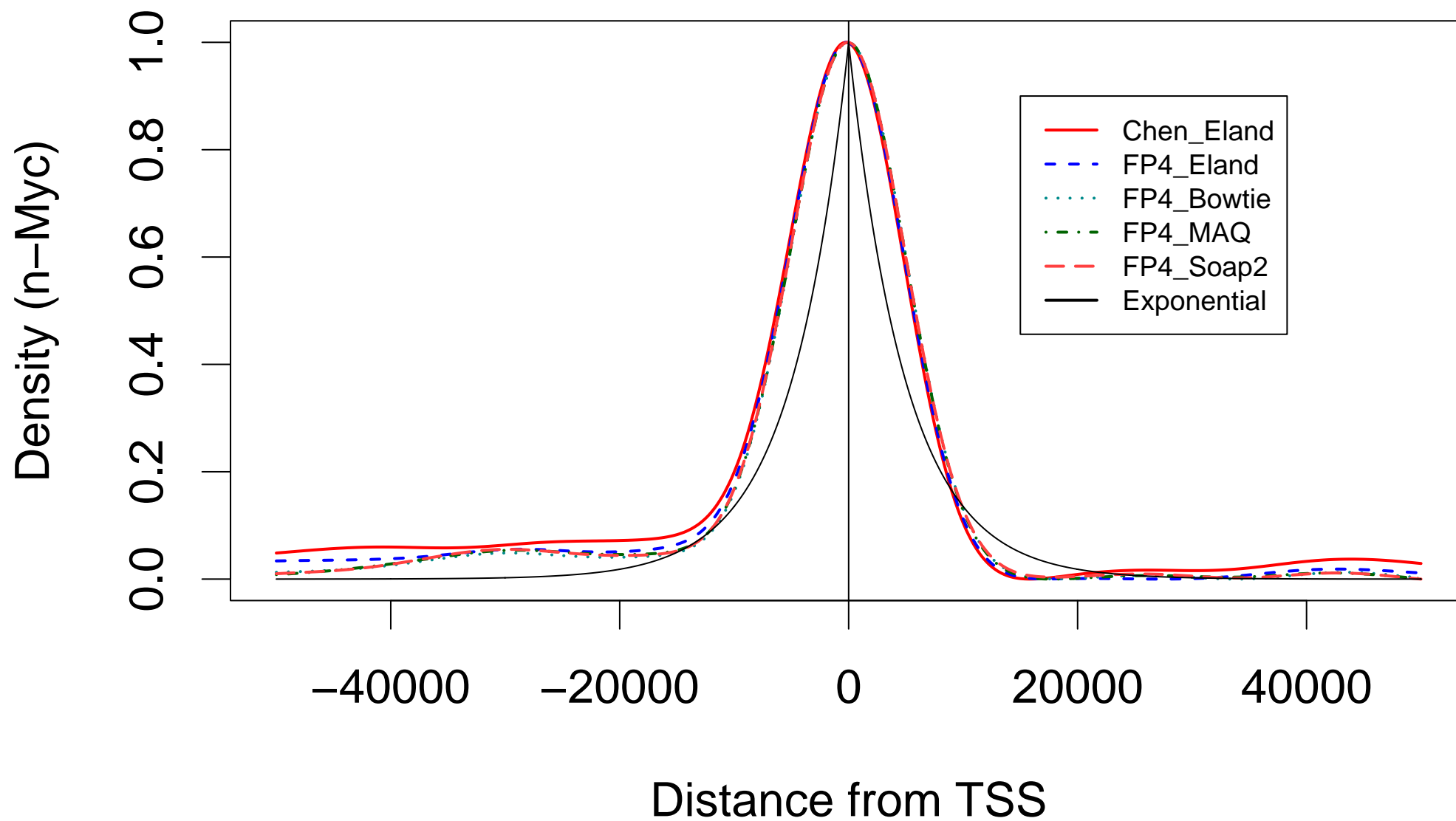

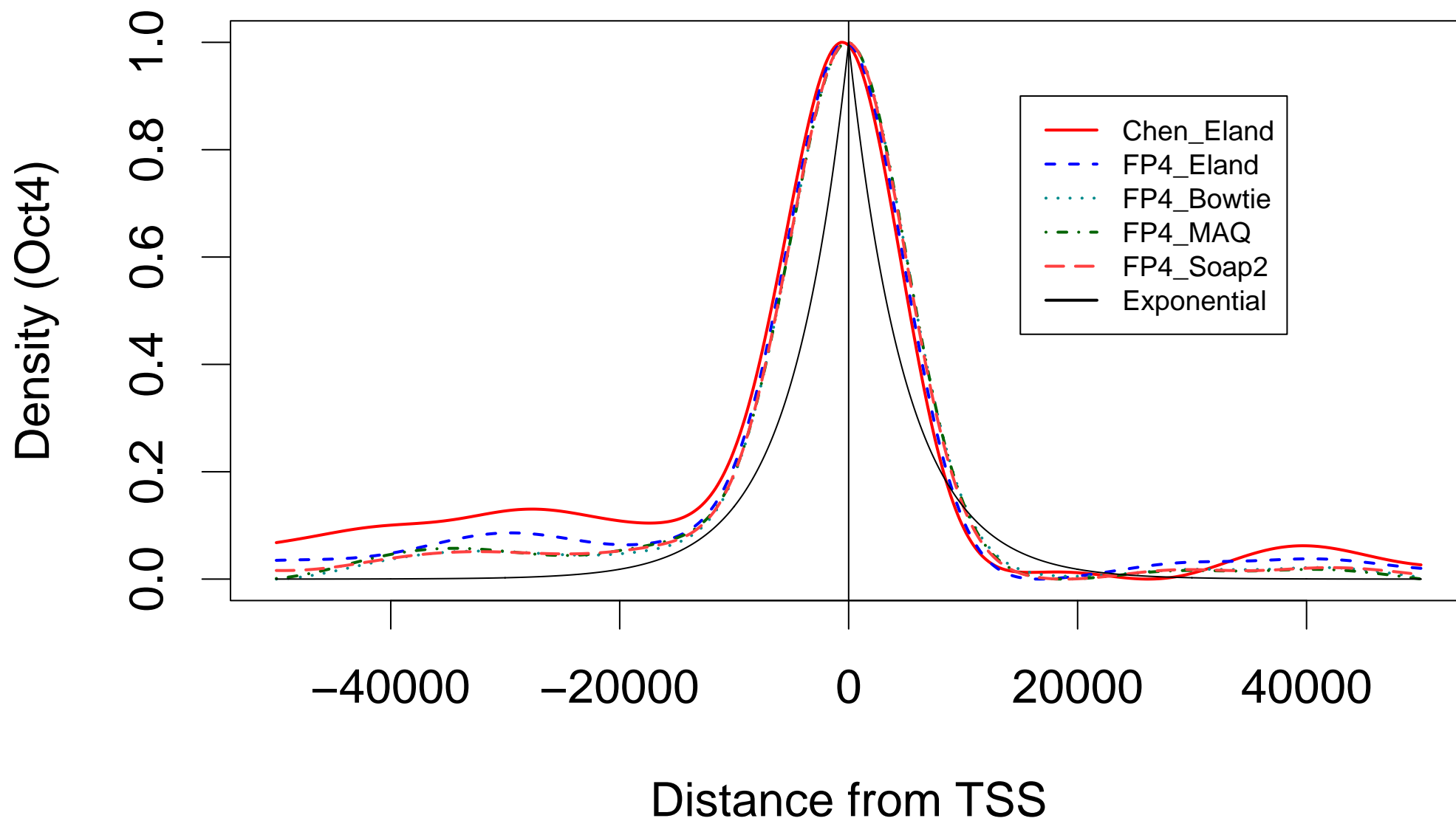

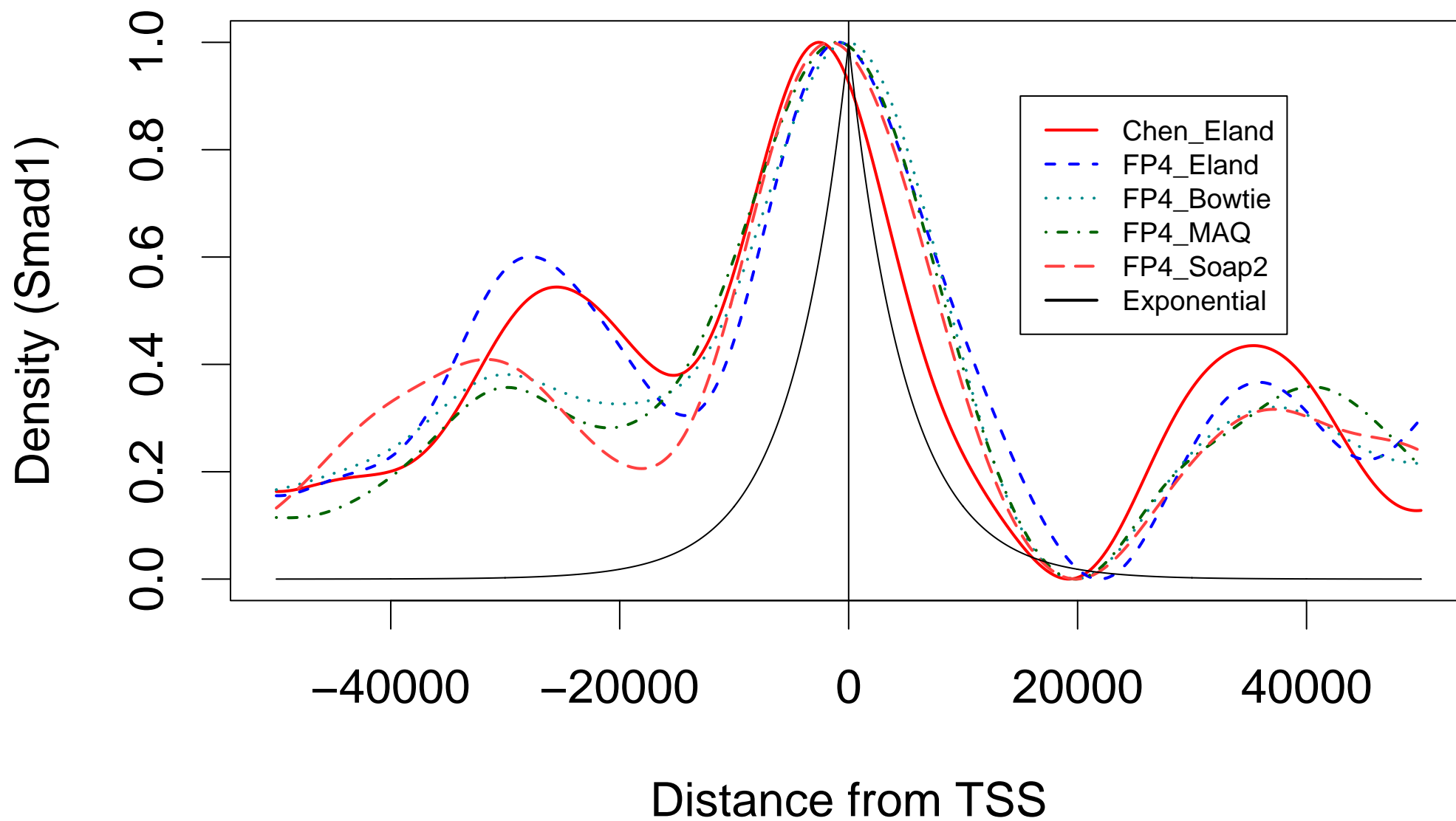

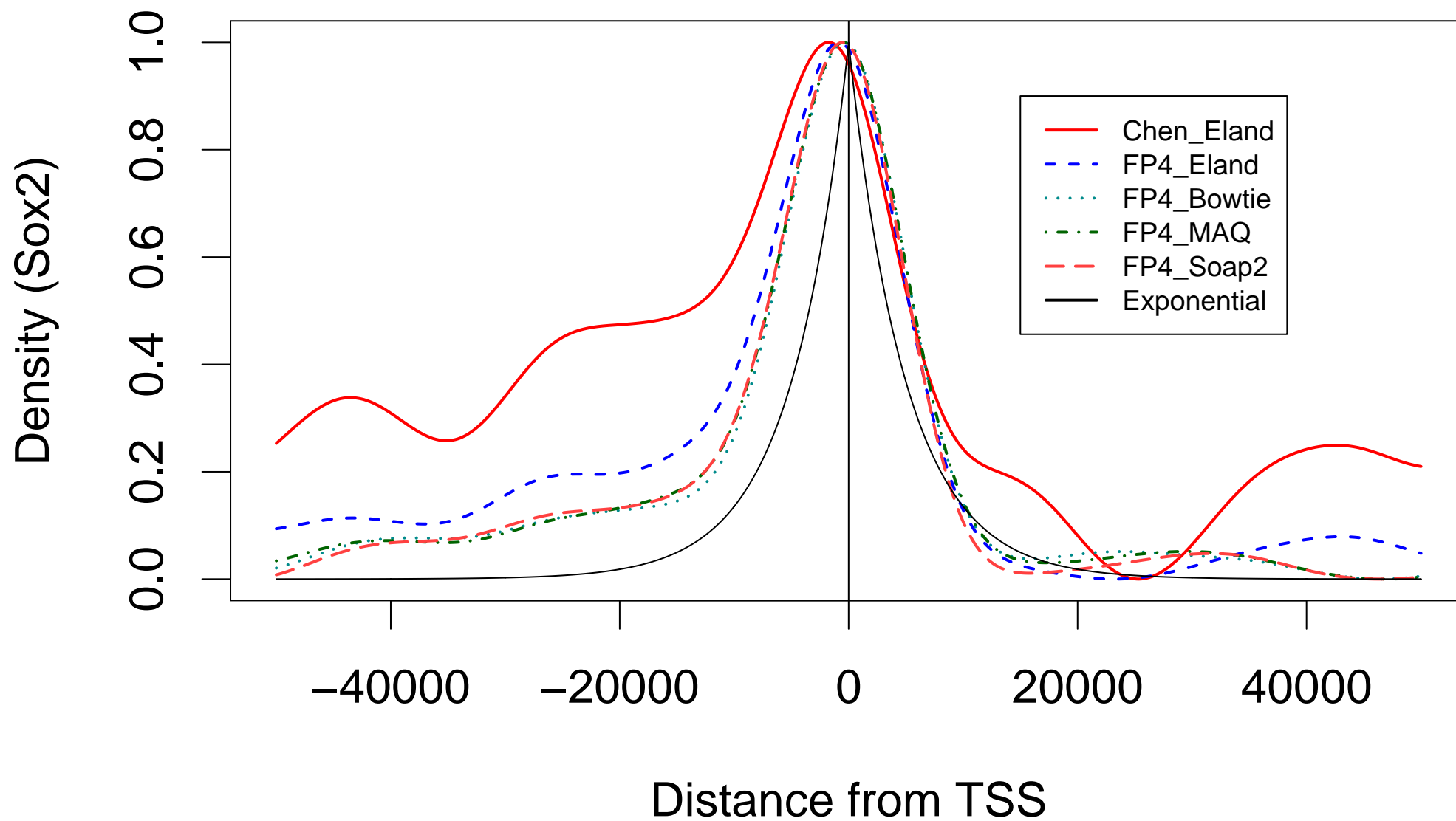

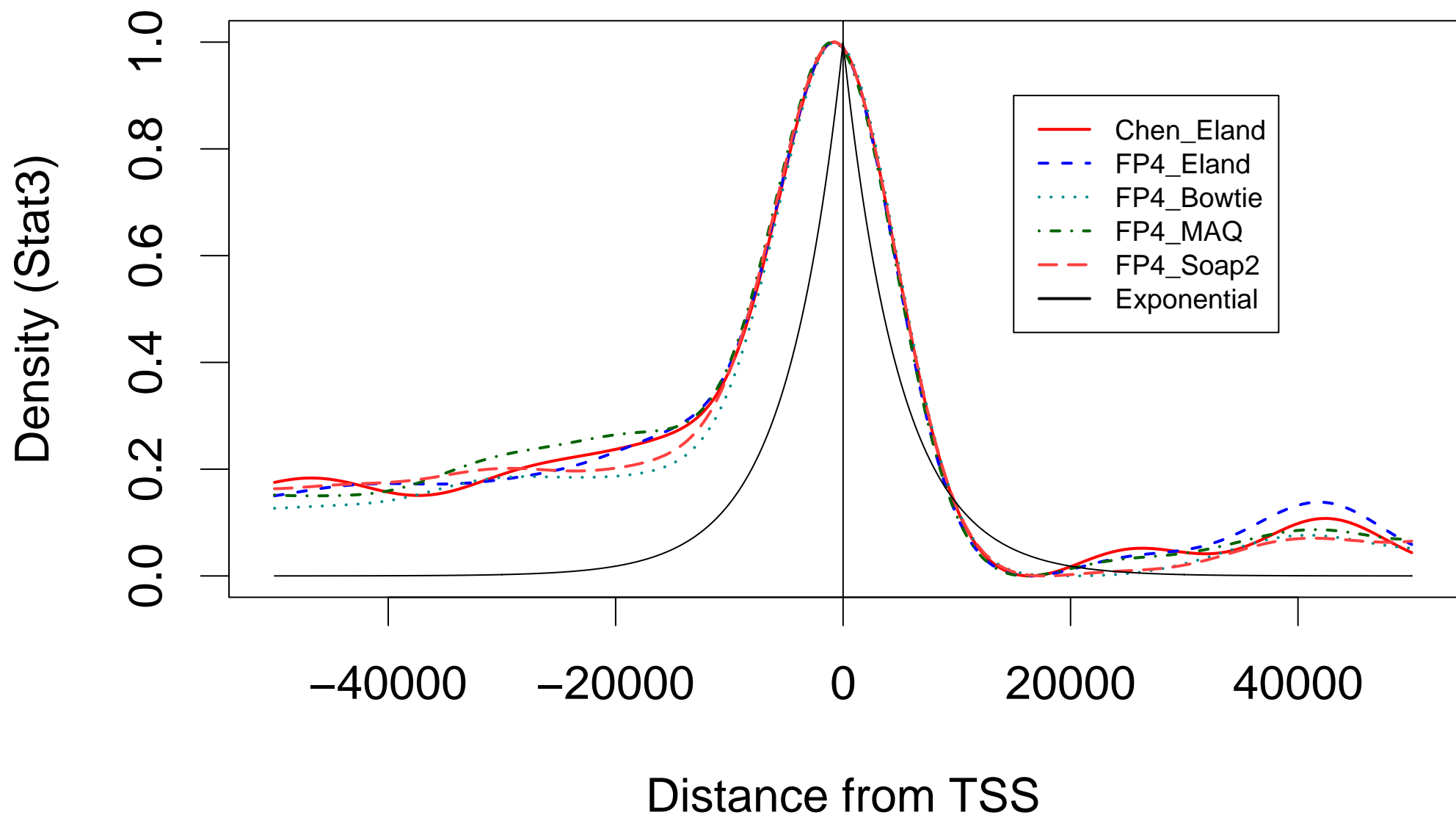

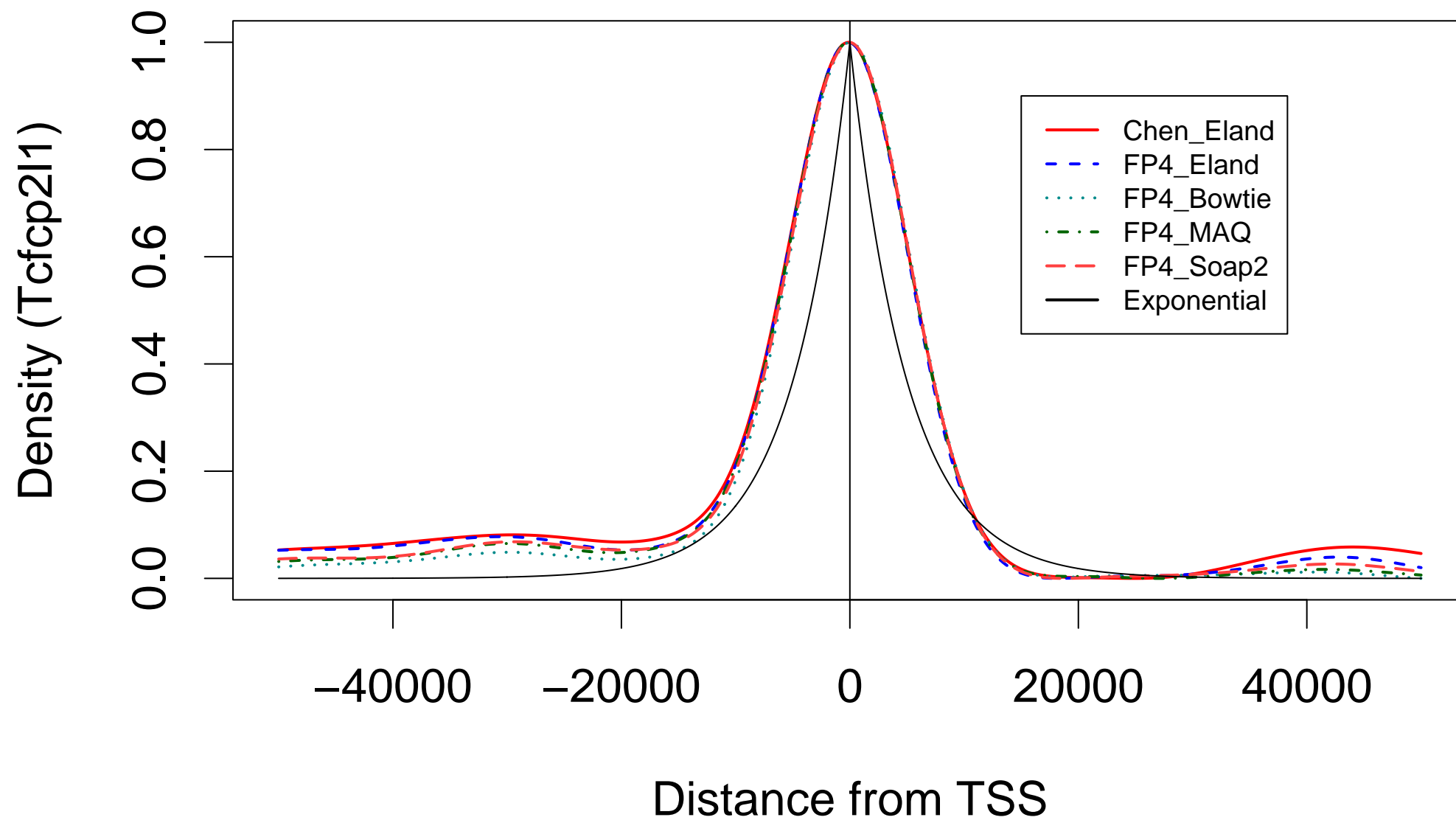

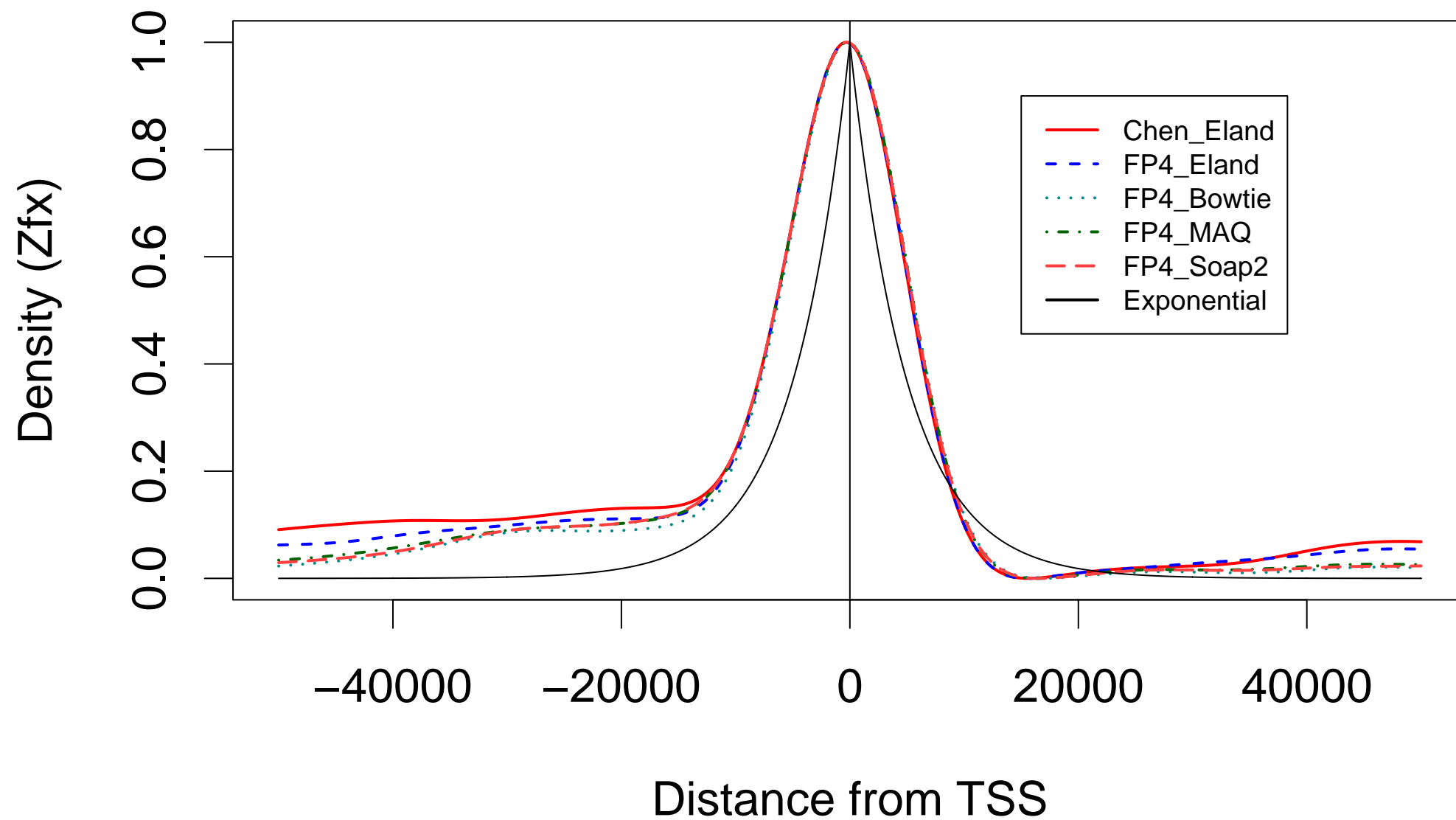

Supplement: Additional file 3 — Density profile of 12 TFs This file provides the density profiles of 12 core TFs in five peak datasets (Figure S2). [file 1471-2105-12-S1-S50-S3.pdf]
